# The Time-Based Effects of Kinesio Taping on Acute-Onset Muscle Soreness and Calf Muscle Extensibility among Endurance Athletes: A Randomized Cross-Over Trial

**DOI:** 10.3390/jcm11205996

**Published:** 2022-10-11

**Authors:** Deepak Malhotra, Shruti Sharma, Ashima Chachra, Meenu Dhingra, Ahmad H. Alghadir, Shibili Nuhmani, Ghufran Jaleel, Raee S. Alqhtani, Mohammed M. Alshehri, Rashid Ali Beg, Mohammad Abu Shaphe, Amir Iqbal

**Affiliations:** 1 Department of Physiotherapy, School of Nursing Sciences & Allied Health, Jamia Hamdard, New Delhi 110062, India; 2 Sports Physiotherapy Department, Stairs Physiotherapy and Fitness Center, Ulsoor branch, Bengaluru 560005, India; 3 Human Performance Lab, Sports Authority of India (SAI), Jawaharalal Nehru Stadium Complex (East Gate) Lodhi Road, New Delhi 110003, India; 4 Rehabilitation Research Chair, Department of Rehabilitation Sciences, College of Applied Medical Sciences, King Saud University, Riyadh 11433, Saudi Arabia; 5 Department of Physical Therapy, College of Applied Medical Sciences, Imam Abdulrahman Bin Faisal University, Dammam 34221, Saudi Arabia; 6 Medical Rehabilitation Department, Medical Applied Science College, Najran University, Najran 11001, Saudi Arabia; 7 Department of Physical Therapy, College of Applied Medical Sciences, Jazan University, Jazan 45142, Saudi Arabia

**Keywords:** KT, sham tape, ankle dorsiflexion, range of motion, pain, muscle soreness

## Abstract

Background: This study aims to determine the effects of kinesio tape (KT) application on acute-onset muscle soreness and the extensibility of the calf muscles in endurance athletes. Methods: A one-arm repeated-measures randomized cross-over controlled study design investigated 55 endurance athletes, including 10 cyclists, 30 badminton players, and 15 long-distance runners (mean age 16.40 ± 2.69) from different stadia in Delhi NCR, India. KT and sham tapes (ST) were applied randomly to right and left legs (prone position) in a cross-over manner with a gap of 72 h. Ankle dorsiflexion range of motion (ADFROM) and pain due to acute-onset muscle soreness were assessed immediately and 10 min and 30 min after treadmill running, using a universal goniometer and numeric pain rating scale (NPRS), respectively, along with the time to fatigue. Results: A statistically significant difference was observed for the NPRS when the mean scores obtained for the KT and ST groups were compared immediately after cessation of running; however, the difference was not statistically significant in the NPRS score that was recorded ten or thirty minutes after. The range of motion increased significantly after the application of both the KT and the ST. After running on a treadmill, the range of motion decreased significantly with both the KT and ST, and the decrease was similar. Conclusion: KT was more effective in reducing the pain intensity immediately after running and increased the time spent running on the treadmill before fatigue set in among endurance athletes. In addition, the two taping methods (KT or ST) were equally effective in enhancing calf muscle extensibility (for both right and left legs) immediately after application. However, both taping methods failed in limiting the decrease in ankle ADFROM after treadmill running.

## 1. Introduction

Acute muscle soreness is caused by local muscle fatigue, which occurs when the muscle is tired and cannot contract any longer [1]. It is associated with a rapid onset of pain within a minute of contraction followed by pain relief within two to three minutes [2] to hours [3,4] after relaxation [2,3,4]. Many sports medicine experts believe that flexibility, which is the ability to move a joint through its complete range of motion, plays a role in injuries whether the injury is due to strains, sprains or overuse [5]. Evidence suggests that muscle stiffness increases immediately after eccentric activity [5,6].

In recent years, kinesio tape (KT) has gained considerable popularity [7]. KT comprises a polymer that is composed of elastic and cotton fibers that has been designed to allow a longitudinal stretch of up to 140% of its resting length. Its thickness is approximately the same as the epidermis of the skin [8]. The suggested benefits of KT include a decrease in pain [1,2], an increase in proprioceptive facilitation [4], muscle facilitation [8], an increase in muscle strength [9], and improvements in pain-free range of motion [10]. KT is also gaining popularity in sports that require repetitive, high-intensity muscular effort and eccentric loading. Several mechanisms describing the action of KT have been proposed following findings indicating that it improves proprioception, enhances muscle performance by strengthening weakened muscles, boosts blood and lymph circulation by removing subcutaneous bleeding or tissue fluid through muscle movement, and alleviates pain through neurological inhibition [8]. Because of its ability to stretch, elastic tape differs from regular cotton, nonelastic tape. This type of stretching stimulates the skin’s sensory receptors, boosts blood and lymphatic circulation, inhibits the neuronal activity of hypersensitive tissue, and realigns joint and fascial tissue [10,11]. These mechanisms might help to improve muscle strength, anaerobic power, and joint stability and help decrease muscle pain [12,13]. The KT is applied to the skin at different tension intensities that vary from paper off to 100% of the maximal stretch of the tape. However, recent work conducted by Chen et. al on the triceps surae muscle during both lying and standing postures indicates that the change in intensity of tension of the KT does not have much effect on the spinal motor neuron excitability; therefore, in terms of modifying sensory-motor activity, the tension applied during a KT application should not be an issue [14,15].

Endurance athletes are those who engage in dynamic sports like jogging, cycling, tennis, hockey, and cross-country skiing that require the sustained usage of vast muscle groups [16], and they commonly use KT for the triceps surae to improve their performance [7]. There is ample evidence for the effectiveness of KT; however, different theories have been suggested for its mechanism of action [17,18,19], and studies have presented contradictory results. Many studies have found KT application to be effective for pain relief [1,2,20] and extensibility [10]. However, many researchers have found that KT does not significantly improve ROM [21,22] and does not have much effect on delayed onset muscle soreness [20,23,24]. There are even fewer studies on the immediate effects of KT. Therefore, our study aimed to determine the effect of KT on acute-onset muscle soreness and the extensibility of the calf muscles at different intervals before and after a fatigue-inducing activity on a treadmill.

## 2. Materials and Methods

### 2.1. Study Design

**Figure 1:**
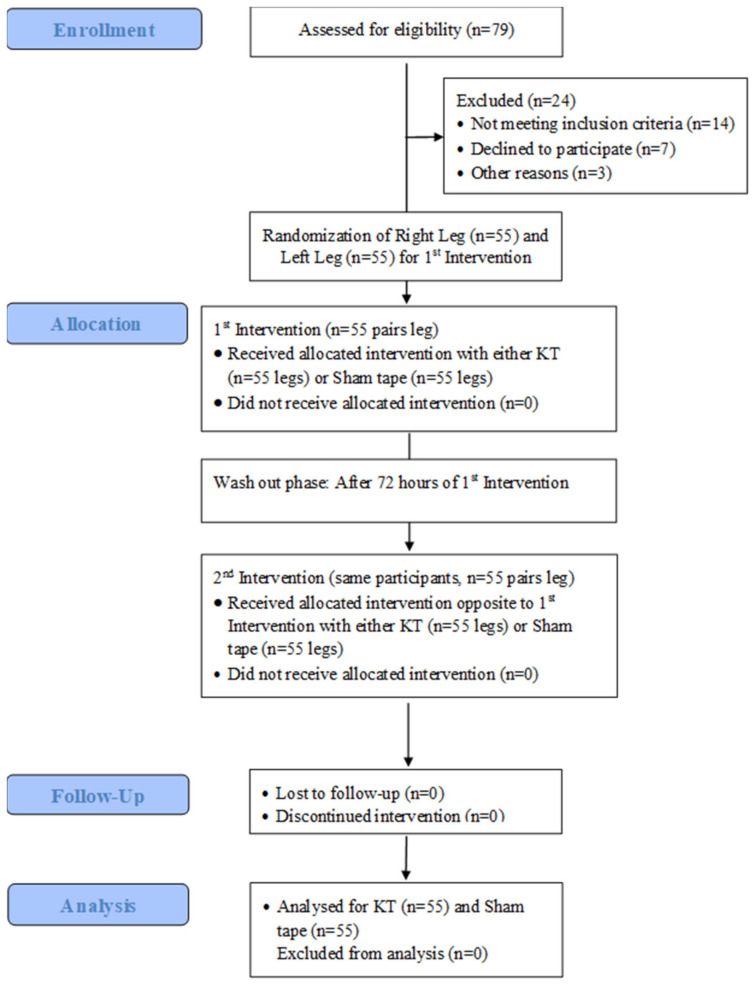
A CONSORT flow diagram for a randomized cross-over trial design depicting the study procedure: enrollment, allocation, intervention, follow-up, and analysis of the study data.

The study was conducted based on a single, blinded, one-arm, repeated-measure randomized cross-over trial design. It was a controlled placebo study and was comparative in nature. The study followed the CONSORT guidelines for a randomized cross-over trial design that guides the repeated application of an intervention in a cross-over manner at two different time points with a washout period for a single group of participants. In the first session, the participants are randomly selected to receive either type of intervention (here, KT or ST). The participants wait till the end of a washout period (72 hours in this study) before repeating the second intervention session. In the second session, a repetition of the interventions takes place with the same participants in a manner opposite to the first session (Figure 1).

#### 2.1.1. Sample Size

The sample size was computed using the effect size (0.43) of the variable pain (VAS scores) from similar studies [13,25], a 0.05 significance level, and a power of 0.80; the required sample size was 45. Considering a dropout rate of 20%, an adequate sample of 55 was determined.

#### 2.1.2. Ethical Consideration

The Jamia Hamdard Institutional Ethics Committee approved this study (ethical approval number: JHIEC-03/18; dated 1530hrs, March 18, 2018); the study followed the Declaration of Helsinki (2013) and was registered on CliniTrials.gov Protocol Registration and Results System with Trial Identifier Number: NCT05564585 (https://register.clinicaltrials.gov; accessed on 29 August 2022). All participants and their legal guardians (in case of minors under the age of 18 years) returned a signed informed consent form before starting the study.

#### 2.1.3. Participants and Setting

A total of 55 endurance athletes (10 cyclists, 30 badminton players, and 15 long-distance runners) from different stadia from the Delhi NCR region of India were included in this study, and the study was conducted over 6 months. The criteria for inclusion in the study were endurance athletes (both males and females) aged between 10 and 20 years with a BMI within the normal range. Participants other than endurance athletes and those who were obese or underweight, who had a history of previous musculoskeletal injuries that could affect performance, and who had either serious medical problems or any skin allergies or open wounds were excluded from the study. The endurance athletes who were included in our study were defined as athletes whose key muscles are exercised at submaximal intensity for a prolonged period. 

#### 2.1.4. Outcomes Measures

Acute-onset muscle soreness-induced pain intensity and calf muscle extensibility were the outcome measures of the study. Pain intensity was assessed using a numeric pain rating scale (NPRS) [1]. The 11-point scale ranges from 0 representing no pain to 10 representing worst pain imaginable [1]. Calf muscle extensibility was assessed as the ankle active dorsiflexion range of motion (ADFROM). The ankle ADFROM was measured using a universal goniometer in a comfortable high sitting posture with the knees at 90 degrees of flexion [20]. The high sitting posture with the knees flexed at 90 degrees relaxes the calf muscles at the knee joint, allowing the full extensibility of the calf muscles at the ankle joint without any limitations. The ankle ADFROM was taken when the participants reached the maximum dorsiflexion by themselves while maintaining thigh contact with the chair. The time to fatigue was also recorded. It was taken as the time until the athlete voluntarily stopped running on the treadmill due to pain (acute-onset muscle soreness) in the calf muscles under different taping conditions.

#### 2.1.5. Procedure

The endurance athletes who had been playing regularly at different stadia in Delhi NCR were contacted individually (one-to-one) for screening and recruitment in the study based on predefined inclusion and exclusion criteria. The participants were informed about the study through individual one-to-one contact, pamphlet distribution, and banners inside the stadium. After completing a signed informed consent form either by the participants or their legal guardians, the recruited participants were assessed for all of the outcome measures. The assessment was completed by a qualified physical therapist in the exercise physiology lab attached to the sports medicine center of the stadium. The height and body mass of the athletes were measured, and each athlete’s BMI was calculated. The ankle ADFROM was measured using a universal goniometer in a preferred posture. After the baseline assessment to determine the ankle ADFROM, each participant ran on the treadmill with both KT and ST on either right or left legs (calf muscle) on two separate days (two sessions), with a gap of 72 hours between first and second intervention session. For the first intervention session, the application of either KT or ST on the right or left legs for each participant was selected randomly, and the tape was applied accordingly. For the second intervention session, the participants received the application of either KT or ST on the opposite leg from the first intervention session. Simple random sampling was applied by having the participants choose chits. All of the participants were blinded to the application method used to apply the tape.

After both types of tape were applied, the 2nd ADFROM measurements were carried out in high sitting posture with the knee flexed at 90 degrees. Each participant was then asked to walk on an inclined treadmill until fatigue was elicited while the participant was wearing the KT or ST. The speed and inclination of the treadmill were set according to the Bruce protocol, starting at 1.7 mph at an incline gradient of 10%, which was increased every 3 min until the athlete reported the onset of fatigue. The treadmill that was used in the current study had an inclination of up to only 15%, so after stage 3, only the speed of the treadmill was increased according to the Bruce protocol [26,27].

After the completion of the treadmill running, the 3rd ADFROM measurements were performed in the same position. Furthermore, each participant was asked to report the presence of any pain in the calf muscles that was due to acute-onset muscle soreness on the NPRS immediately after the treadmill running as well as 10 min and 30 min after.

A CONSORT flow diagram for a randomized cross-over trial design depicting the study procedures, including the enrollment, allocation, follow-up, and analysis, is presented in Figure 1.

#### 2.1.6. Interventions

##### Kinesio Tape Application

**Figure 2:**
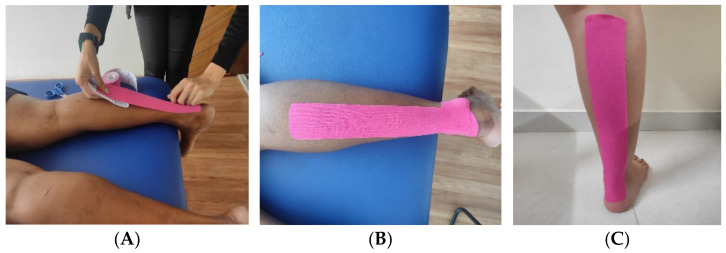
KT and ST applications: (**A**) KT application step1; (**B**) KT application step2; (**C**) ST application.

For KT application (3NS TEX, Kinesiology Tape, Hitech Therapy, Randburg, SA), the I-shaped taping technique was used in which there are two strips, one used as an anchor and the other as a functional strip [21]. The width of either strip was 5 cm, while the length of the functional strip and anchor strip was per each participant’s calf muscle length and width. The base of the functional strip was applied distal to tendo-achilles insertion without any stretch to the tape with ankle neutral. After the application of the base, the participants were asked to perform dorsiflexion, and the functional strip was applied to the stretched muscle while maintaining a 10% tape pre-stretching. Lastly, the anchorage was applied without stretching, just proximal to the insertion of muscle with ankle neutral [21]. The KT was applied with the subject in prone position, and all KT applications were managed by the same investigator to maintain the consistency of application (Figure 2A,B). The medical acrylic-based adhesive is used in the KT [28].

##### Sham Tape Application

For the ST application, small pieces of the same elastic tape were loosely applied to the calf muscle per the KT application procedures except for the functional strip. The functional strip was applied to the calf muscle in a non-stretch position (i.e., without active dorsiflexion) (Figure 2C).

## 3. Data Analysis

Data were analyzed using SPSS 17.0 software (Chicago, IL, USA). The outcome scores for the ankle ADFROM and the NPRS were considered for the analysis. A Shapiro–Wilk test of normality was applied for examining the distribution of the continuous variables. A paired *t*-test was used to compare the differences in the range of motion before and after intervention and after treadmill running for both the KT and the ST, and differences in the NPRS after the application of the KT and ST were observed. The tests were applied at the 95% confidence interval, and the level of significance was set to 0.05. Results were taken to be significant if *p* < 0.05.

## 4. Results

Initially, a total of seventy-nine participants were screened, and fifty-five participants were recruited for the study. A Shapiro–Wilk test revealed a homogenous or normal distribution (95% CI, *p* > 0.05) of data in this study. The demographic characteristics and baseline measurements of the participants kept in a single group are presented in Table 1.

### 4.1. Pain

Mean NPRS scores that were obtained immediately and 10 min and 30 min after the fatigue-inducing activity on the treadmill were compared between the taping conditions (Table 2). The results showed a statistically significant difference in the mean NPRS score between the two groups immediately after completion of the activity, but this difference was not statistically significant in the NPRS score that was recorded 10 min after the completion of the activity. Approximately 30 min after the activity, the participants were pain-free after the use of both the KT and the ST; therefore, this was not analyzed and is not presented in the Table 2.

### 4.2. Extensibility

**Figure 3:**
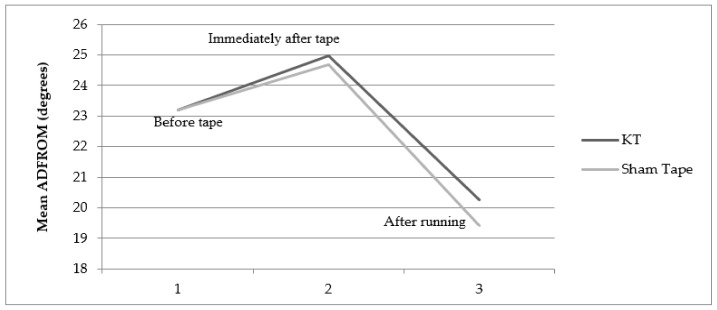
Change in ankle active dorsiflexion range of motion with the KT and ST in the right ankle.

**Figure 4:**
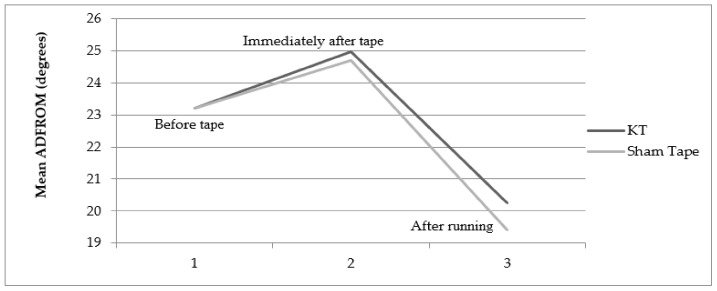
Change in ankle active dorsiflexion range of motion with the KT and ST in the left ankle.

The mean dorsiflexion range of motion was compared for different taping conditions for both legs—before the tape was used and immediately after the tape was used, as well as immediately after the tape was used and after running (Figure 3 and Figure 4). The range of motion (degrees) increased significantly immediately after the application of both the KT (23 ± 3.75° to 25.13 ± 4.146°, *p* = 0.01 in the right leg; 23.20 ± 4.057° to 24.98 ± 4.089°, *p* = 0.01 in the left leg) and the ST (23.22 ± 3.75° to 24.6 ± 3.804°, *p* = 0.01 in the right leg; 23.20 ± 4.057° to 24.69 ± 3.776°, *p* = 0.01 in the left leg) compared with before the application of the tape. However, after running on the treadmill, the range of motion decreased significantly with the application of both the KT and ST in both the right and left legs compared with immediately after the tape used (i.e., within-group effect). Though the decrease in ankle ADROM in the right and left leg was smaller with the KT compared with the ST (i.e., between-group effect) immediately after running, the difference was not statistically significant (*p* = 0.45 for right and *p* = 0.34 for left).

### 4.3. Time

**Figure 5:**
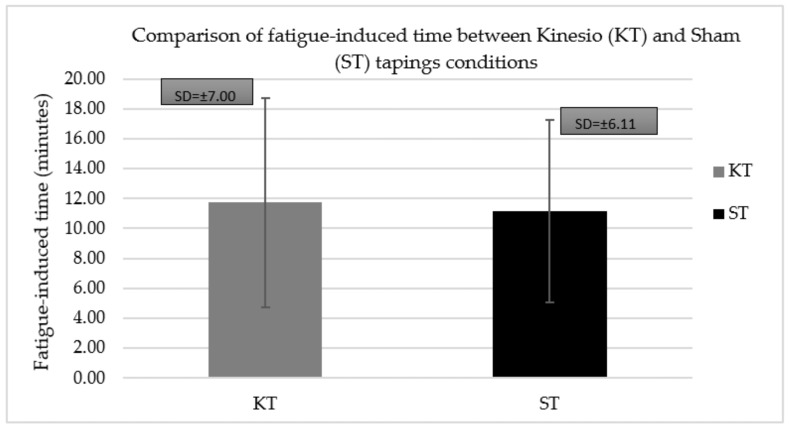
Comparison of fatigue-inducing time for which athlete could run on treadmill after the application of KT and ST.

The time spent running on the treadmill before fatigue set in was also recorded and compared between the different taping conditions (Figure 5). The results showed a statistically significant difference (*t* = 1.98; *p* = 0.05) between the mean time for which the athletes could run before fatigue set in for the KT (11.74 ± 7.00 min) and ST (11.14 ± 6.11 min) conditions.

## 5. Discussion

The study aimed to determine and compare the effects of KT and ST on pain intensity caused by acute-onset muscle soreness and the extensibility of the calf muscles among different types of endurance athletes, specifically cyclists, badminton players, and long-distance runners. The scores for the NPRS and ankle ADFROM outcomes were evaluated within and at different time intervals between the different conditions (KT and ST). The findings of this study indicate that the application of KT may decrease the pain caused by the acute onset of calf muscle soreness developing after fatigue-inducing activity on a treadmill, at least immediately after the cessation of running activity. However, it does not affect the extensibility of the calf muscles after an acute bout of endurance activity. The pain that was reported by the athletes after the completion of fatigue-inducing activity on a treadmill after KT application was less than that reported after ST application. Some studies have reported the promising effect of KT on pain relief [1,21,22,23,24,25,26,27,29]. However, a systematic review study declared that the KT should be considered as an adjunct or complementary therapy rather than the leading treatment choice for pain relief [20]. In contrast, some studies have shown that KT is not effective for pain reduction [5,30,31,32]. The reason for those contradictory results could be that the samples from these earlier studies were acquired from among injured individuals, whereas in this study, the pain was post exertional. In a similar study, Merino-Marban et al. found that KT effectively reduced post-exertional pain in duathlon athletes [23]. 

Taping’s mode of action is unknown, and the physiological mechanisms that have been postulated are speculative. However, it is thought to operate by delivering continual proprioceptive input or by correcting alignment while movement is taking place [32,33]. Gate control theory is also one of the explanations that have been proposed to explain the reduced pain. It has also been suggested that taping stimulates neuromuscular pathways, resulting in increased afferent feedback [34]. Space correction and lymphatic effects are also probable explanations for the immediate effect of KT. When applied with minimal tension, KT creates convolutions under the skin. This widens the space under the skin and in turn allows fluid to move away from and into the affected area, promoting the healing of injured tissue [8]. When KT has been applied with a very light stretch (about 20%), the skin, fascia, and muscles are stretched from insertion to the muscle’s origin along its alignment. This, it is said, causes an eccentric pull on the fascia and muscle, inhibiting muscle contraction and thus facilitating healing and pain relief [3,9,35]. It has also been proposed that KT has a mechanical effect that results in pain reduction. The mechanical forces of KT may help to alleviate fascia stiffness, which can occur during acute inflammation or when the fascia hardens and loses flexibility [3,36]. 

This study also found that athletes could run for a longer amount of time when KT was applied than when the ST was applied. The reason for this finding could be decreased post-exertional pain when KT is applied. It has also been proposed that the KT supports damaged structures while simultaneously allowing mobility. It is also believed to influence blood flow and proprioception, which are associated with muscle fatigue [37]. Some studies have shown improvements in muscle endurance with the application of KT. Lindsey et al. found that elastic tape improved back muscle endurance compared with in the no-tape condition [38]. The effect of KT on back muscle fatigue was studied, and the authors found that KT delayed the onset of muscle fatigue [37]. Osorio et al. also found that patellofemoral taping increased knee muscle endurance in patients with patellofemoral joint pain [39]. In a placebo-controlled trial, Cowan et al. found that the application of KT altered the activation of VMO and VL during stair climbing in patients with patellofemoral pain syndrome [40].

In this study, there was a significant increase in the dorsiflexion range of motion after both the KT and the ST were applied. After running on the treadmill, the range of ankle dorsiflexion decreased on both occasions. Although the decreased range of motion was reduced when the athletes ran with KT, the difference was not statistically significant. Many studies have found that KT has a positive effect on muscle extensibility [8,10,41,42,43]. However, most of these studies were conducted on injured individuals and did not have control groups. In line with this study, many studies have found KT application to be ineffective in improving extensibility [21,22]. The initial effects of KT on trunk flexion were investigated by Salvat and Alonso [22]. They randomly assigned 33 people to one of 3 groups, KT, standard tape, or placebo, and found no statistically significant differences in sit-and-reach scores [22]. Merino-Marban et al. investigated the acute effects of KT on hamstring extensibility and discovered no statistically significant differences between the KT and placebo groups [21]. 

Many mechanisms have been proposed to explain how KT improves muscle extensibility. One theory is that elastic stretch tape directly exerts tension in the direction of insertion of the muscle to the fixed base and displaces the skin in the same order. Thus, elastic stretch tape causes a reduction in muscle contraction to a certain point by keeping the same muscle in an extended position, allowing the antagonistic muscle to contract more to increase the ROM simultaneously [42]. Another theory is that the KT stimulates the underlying mechanoreceptors that contribute to the increasing range of motion [21]. One more theory advocates that KT brings physiological changes that may alter the myofascial and muscle functions by improving the blood flow to the underlying taped area [21,22].

## 6. Limitations

There was a great variability in the ages (participants were 10 to 20 years of age) and types of activities carried out by the athletes—10 cyclists, 30 badminton players, and 15 long-distance runners—who participated in this study, which could have affected the outcomes due to differences in the flexibility and endurance among children and adults and types of the activities carried out in different games. The levels and types of activities the athletes were playing and their experiences varied, affecting their endurance. Due to prior exposure or experience with KT, it can be difficult to control subject bias, even when blinded to the taping settings. As the sample size was considerable but not very large, there are chances of Type II errors being present in the results. Moreover, the speed and inclination of the treadmill were set according to the Bruce protocol, starting at 1.7 mph at an incline gradient of 10%, with increases in speed and inclination every 3 min until the athlete reported the onset of fatigue. However, the treadmill that was used in the current study had an inclination of up to only 15%, so after stage 3, only the speed of the treadmill was increased according to the Bruce protocol. This discrepancy might have affected the time and amount of exertion reported by participants who developed the acute onset of muscle soreness during treadmill running. 

## 7. Practical Application of the Study

Since the results show that application of the KT positively affects the time to fatigue and the perception of pain in the calf muscles after an endurance running activity, we conclude that the application of KT tape over the calf muscles would help to prolong the activity before the athlete starts experiencing pain. Prolonged activity time involving endurance running should culminate in enhanced performance in sporting activities. This however should be permitted by the rules of the game if the athlete is taking part in a formal competition and has to be empirically tested with appropriate research designs.

## 8. Scope for Future Studies

The effects of KT need to be studied on a larger group of athletes with more homogeneity regarding the age and experience of the participants. The immediate effects of KT on muscle strength and power also need to be researched.

## 9. Conclusions

Kinesio taping was more effective in reducing the pain intensity and delaying the fatigue-inducing time caused by acute-onset muscle soreness in calf muscle than sham taping immediately after treadmill running among endurance athletes. In addition, the kinesio taping and sham taping were equally effective in enhancing calf muscle extensibility (for both right and left legs) among endurance athletes after immediate application. However, both kinesio and sham taping failed to limit the decrease in ankle ADFROM and to reduce the pain intensity 10 min after treadmill running. KT was effective in reducing the pain intensity immediately after the fatigue-inducing activity on treadmill. Further, clinical trials are required to investigate the long-term efficacy of tapping on pain modulation and muscle repair following soreness.

## Figures and Tables

**Table 1:** Demographic statistics of the participants kept in a single group (*N* = 55).

Variables		Mean ± SD	Minimum	Maximum
Age (years)		16.36 ± 2.69	10	20
Gender	Male	31	n/a	n/a
	Female	24	n/a	n/a
BMI		19.44 ± 1.14	16.40	21.39
ETE (years)		1.95 ± 0.91	1	5
FT (min.)	KT	11.74 ± 7.01	4.20	30
	ST	11.14 ± 6.11	4.34	30
ADFROM (°)	KT (Lt/Rt)	23.20° ± 4.057°/23.20° ± 3.75°	15°/18°	42°/40°
	ST(Lt/Rt)	23.20° ± 4.057°/23.22° ± 3.75°	15°/18°	42°/40°

SD: standard deviation; ETE: endurance training experience; FT (min.): fatigue-inducing time in minutes; KT: kinesio tape; ST: sham tape; ADFROM: ankle dorsiflexion range of motion; NPRS: numeric pain rating scale; °: degree sign.

**Table 2:** Time-based comparison of NPRS scores immediately after and 10 min after the completion of the fatigue-inducing activity on a treadmill under both Kinesio and Sham taping conditions, using a paired *t*-test.

Variables	With Kinesio Tape		With Sham Tape		Paired *t*-Test	
	Mean (cm)	SD	Mean (cm)	SD	*t* value	*p*-Value
NPRS0	3.28	1.257	3.89	1.165	2.388 *	0.020 *
NPRS10	1.04	1.071	1.24	0.922	1.466	0.154

*—Significant at 0.05 level; NPRS0: NPRS scores immediately after completion of the fatigue-inducing activity; NPRS10: NPRS scores 10 min after completion of the fatigue-inducing activity.

## Data Availability

The data set for the result of this study will be available from the corresponding author upon reasonable request.
